# Understanding the Experiences of Designated Assessors: an Interpretive Phenomenological Study

**DOI:** 10.1007/s40670-026-02724-5

**Published:** 2026-04-17

**Authors:** Elizabeth B. Bradley, Cayla R. Teal, Andrew S. Parsons, Maryellen E. Gusic

**Affiliations:** 1 Department of Medical Education, University of Virginia School of Medicine, PO Box 800866, Charlottesville, 22908 VA USA; 2 Office of Medical Education, University of Kansas School of Medicine, Kansas City, KS USA; 3 Department of Medicine, University of Virginia School of Medicine, Charlottesville, Virginia USA; 4 Educational Affairs, Medical University of South Carolina College of Medicine, Charleston, SC USA

**Keywords:** Designated assessors, Entrustable professional activities, Competency-based medical education

## Abstract

**Introduction:**

The expanded use of Entrustable Professional Activities in medical education has led to a call to address concerns about implementation challenges and the validity of this framework for assessment. The use of well-trained, designated assessors (DAs) who not only provide feedback to learners but also participate in summative decision-making about learners’ competency is one approach to address these issues. The lived experience of faculty who serve in this role within a program of assessment, including their beliefs and practices, has not been described in the literature. This study examines the experiences of designated assessors at a single institution, concentrating on their dual roles as formative assessors and summative decision-makers.

**Methods:**

The experiences of designated assessors were explored using an interpretivist phenomenological approach. All DAs at one institution were invited to participate in the study. Interviews were recorded and transcribed verbatim. The interview guide was designed to investigate various aspects of the DA experience. An iterative process of content analysis, careful consideration of participants’ statements, and memoing ultimately yielded three themes.

**Results:**

Six of eight eligible participants were interviewed. Three themes were developed from in-depth interpretation, which inform the designated assessor’s experience. These interpretations center on role enactment, community meaning-making, and the identification of struggling learners.

**Discussion:**

Designated Assessors provided rich descriptions of their processes, shared experiences, and meaning they derive from these experiences. The DAs experience the unique assessor community they are building, the consequential nature of the assessments, and the high-quality assessment data they provide as valuable to learners and those who support them.

A program of assessment that includes Entrustable Professional Activities (EPAs) incorporates direct observation, personalized and timely feedback, the use of multiple assessments to gather data, and an evidence-informed system of decision-making about learner advancement based on entrustment [1]. The expanded use of an EPA framework for workplace assessment within Competency-Based Medical Education (CBME) programs has come with concerns about the validity, reliability, and use of data for learner feedback and summative decision-making [2–4]. Tensions regarding clarity around the purpose of assessment, the role of assessors, and the use of data have led to suggestions that faculty be identified for distinct responsibilities within a program of assessment [5, 6]. Challenges associated with successful implementation include ensuring that faculty assessors have dedicated time to participate in professional development related to a new assessment framework and to protect time to observe learners’ performance in the context of busy clinical environments [6–9]. The use of designated assessors is one approach to address these practical hurdles.

Well-trained, competent assessors who are committed to and understand the purpose of the assessment program, the criteria being applied, and the implications of decision-making support the reliability and acceptability of an EPA-based assessment program [10]. Specifically trained “designated” assessors (DA) who conduct frequent, context-neutral, expert assessments provide a mechanism for obtaining high-quality, workplace-based assessments that produce actionable feedback for learners [11, 12]. Potential benefits of incorporating designated assessors into a program of assessment may include minimizing bias, separating the roles of assessor and clinical supervisor, and enhancing learner skill development by consistently applying performance expectations to provide feedback [13–15].

Another foundational component of EPA assessment is the integration of data from these assessments into a “robust system for decision-making” [16] about learners’ competence. This is often addressed by a competency committee (CC) that includes members with diverse ranks, academic foci, gender identities, racial/ethnic identities, teaching and assessment experience, and other relevant characteristics [17]. CC members must undergo purpose-driven, consistent training to support their development as decision-makers. Skill-building efforts focus on developing a shared mental model of assessment purpose, the ability to analyze EPA and other assessment data against clearly stated performance expectations, and a solid understanding of group decision-making processes and expectations [18–21]. Implementing processes and structures that enable the collective analysis and synthesis of learner assessment data, including EPA assessment data, is a step toward addressing the challenges posed by high-stakes decision-making [1, 9, 10, 22, 23]. Designated assessors, as members of a competency committee, could bring a unique and important perspective, given their first-hand experience in applying the assessment criteria while directly observing learners’ performance across a variety of contexts.

The beliefs, practices, and experiences of faculty who serve as designated, frequent assessors remain unexplored. In this study, we explore the following question: “What are the lived experiences of the designated EPA assessors at our institution, focusing on their dual roles as formative assessors and summative decision-makers?”

## Methods

### Design

To effectively address our research question, we employed an interpretivist phenomenological approach, which focuses on understanding participants’ lived experiences and how they make sense of these experiences within their own contexts [24, 25]. This approach, situated in our belief that knowledge is socially constructed [26], seeks to illuminate the meaning structures embedded in participants’ words through iterative, interpretive engagement with the data and provides a mechanism for understanding and interpreting the potential uniqueness of the designated assessor role. This study was approved by the UVA Social and Behavioral Sciences IRB #4158.

### Reflexivity

Our research team included four individuals, each with training and experience in qualitative research methodologies, including phenomenology. Two researchers were inaugural members of the EPA Leadership Team, which planned and developed the program (MG and EB). Three were heavily involved as DA program administrators (MG, AP, and EB) and were very familiar with the individual DAs, having worked closely with them as colleagues. MG and AP are clinician educators. EB oversees program evaluation. In keeping with an interpretative phenomenological approach that understands the researcher as part of the context rather than separate from it, it is important to note that the researchers were experienced in assessment, the EPA program [1], and the broader context of the DA experience. All aspects of this study were therefore filtered through these experiences. A fourth team member was a professional colleague from another institution (CT) with extensive experience in assessment and qualitative research, and general familiarity with the DA program (though no familiarity with the actual DAs). This individual conducted all interviews and managed all communication with participants, ensuring that they could speak freely and without influence from EPA program leadership. Throughout the analysis, the two primary analysts (EB and CT) reflected upon their roles (as a DA program leader and as the interviewer, respectively), by memoing and regularly discussing how their roles and experiences influenced the development of the codes and categories.

### Setting

The University of Virginia (UVA) designated assessor program directly addresses two practical challenges related to implementation of workplace-based assessment (lack of dedicated time and ongoing professional development). Designated assessors are selected for this 0.20 FTE role based on their experience as experienced clinical educators. The assigned FTE support enables DAs to focus solely on their EPA assessments when scheduled with students. They participate in regular professional development to ensure that their EPA assessment skills are standardized and that their assessment data are of high quality. The DAs are “context-neutral,” providing workplace-based assessments during actual patient encounters across a variety of clinical contexts and disciplines. Best practices for direct observation and actionable feedback are foundational and reinforced topics in the professional development sessions. In addition, as members of an Entrustment Committee (EC), the designated assessors work together using a shared mental model they created and continue to refine to apply performance expectations and ensure consistency in their analysis of assessment data across students and assessors.

This program assesses the following AAMC Core EPAS for entering residency: EPAs 1 (history and physical exam), 2 (differential diagnosis), 3 (diagnostic and screening tests), 4 (orders and prescriptions), 5 (written note), 6 (oral presentation), 7 (clinical questions and use of evidence) [27]. At least 12 times during the clerkship and post-clerkship phases, all students initiate required EPA assessments with DAs in all required clerkships and three required post-clerkship courses, utilizing a web/app-based scheduling tool. Students are instructed to do the assessments as part of their daily care with the patients for whom their clinical team is responsible. They can work with each DA a maximum of two times during each phase. Two hours are allowed for a DA to observe, assess, and provide feedback to students, though time spent varies based on the complexity of the patient encounter and the EPA(s) being assessed. Within 72 h of the observation, DAs use a web-based form to provide the student with a prospective supervision rating and verbal and written feedback to support this rating [28]. Students can access the results as soon as these data are entered. The results are also available to the student’s clinical coach [29]. At regular intervals during the clerkship and post-clerkship phases of the curriculum, the group of DAs meets as an EC to discuss student performance to date and provide written feedback based on an analysis of aggregated data. These discussions culminate in recommendations made to the School of Medicine’s Advancement Committee about each student’s readiness to progress in the curriculum and assume additional responsibility in patient care.

### Interviews

In-depth, semi-structured interviews were selected for data collection to gather detailed descriptions of participants’ experiences through conversational interactions [30]. At the time of the study, eight DAs from surgical and non-surgical clinical disciplines were working in the program; all were experienced medical educators and expert clinicians and were invited to participate. The initial interview guide was developed by adapting Bevan’s approach for use with our interpretivist phenomenological lens [31]. This more flexible guide allowed probing and follow-up questions to support deeper interpretation of meaning and context. The interview guide was piloted with a faculty member who had recently been trained to serve as a DA, had conducted a few student assessments, and had participated in the EC but only as a training exercise. After the pilot, the interview guide was revised slightly to clearly outline the interviewer’s specific process and ensure a smooth flow from question to question. This final guide was structured to explore how and why participants chose to be DAs, their day-to-day experiences as DAs, and how they make meaning of their DA experiences. Questions also explored the dual roles of the DAs – assessing students and working together as the EC. The interviewer followed the participant’s lead on how and when these were explored. In-depth, loosely structured interviews were conducted virtually by the non-affiliated team member (CT). The interview guide is available upon request.

Each interview was recorded and transcribed verbatim using a commercial transcription service. Each participant was allowed to review their transcript and correct any incorrectly transcribed text. Per our IRB approval, we created some participant ambiguity by asking a colleague who no longer worked at the institution to de-identify each transcript to protect interviewees’ identities. We removed all references to participant names, departments, specialties, and any other identifying information. The transcripts were stored securely in a folder available only to the interviewer (CT) and the colleague involved in de-identification. Once de-identified, the interviewer made the de-identified transcripts available to other team members and destroyed the original recordings and identifiable transcripts.

### Analysis

Considering the general approaches to data analysis suggested by van Manen [30], and more conventional approaches to detailed qualitative analysis [26], we immersed ourselves in the data as it was collected, reflecting and memoing our understandings. We engaged in close, iterative reading of transcripts. While we initially labeled segments of text to aid organization, our analytic process was guided by van Manen’s selective highlighting approach, attending to statements that illuminated essential aspects of the lived experience. Coding served as an organizational tool rather than a categorical reduction of data. Four early considerations emerged that framed the analysis and facilitated our initial understanding. These were: the DA’s motivation, their processes, the value and meaning of the experience, and any existing role tensions. Using Dedoose [32] to manage organizational coding, two team members (EB and CT) independently read and coded each transcript using in vivo and open coding. Each analyst also wrote memos for each transcript, reflecting on their understanding of the interviewees’ experiences and capturing their initial impressions of the overall experience and its meaning. EB and CT discussed the codes and memos, resolved the final codes, and documented the overall interpretation of each transcript [33]. One team member (MG) periodically reviewed and corroborated interval findings.

These processes (i.e., creating an audit trail through memoing and other discussion documentation, reflection on researcher bias, iterative review of codes and categories, and verification by the third team member) helped establish the trustworthiness of the data. In addition, we sought participants’ perspectives on the findings (i.e., conducted a member check [34]) and asked them to verify and provide suggestions for clarification.

## Results

Six of the eight eligible DAs agreed to participate in the study. Participants were professors or associate professors from both non-surgical and surgical disciplines and identified as male or female.

Analysis and interpretation of interview text and the researchers’ reflective and descriptive memos yielded nearly 350 statements that, in our view, illuminate the lived experience of being a designated assessor. See Table 1 for examples of these statements. From these data, we generated three themes: role enactment- what the DAs do and why; community meaning-making- how a shared assessor identity and language are crafted; and how DAs experience the work of identifying struggling learners. Below, we describe the DA’s lived experience in their own words, with quotations identified by unique participant identifiers.

**Table 1:** Examples of participant statements that served as organizational codes

• "then 10% on each end most difficult"	• "a lot to look at" in indiv/pair review	• DA program is "messy, full of gray"
• "fruitful and developmental encounter"	• "getting over the hump of hurting people's feelings"	• "comfort, confidence and skill"
• "experience helps teach"	• "grading would be harder"	• "finds it rewarding"
• "fair as possible"	• "hardest to keep mouth shut"	• "disappointment" if no DA program
• "finding root cause" of student's poor performance is key	• "individual/pair review is boring"	• "try to avoid biasing myself"
• "not upset or hurt patient"	• "lots of time" (neg)	• "authentic work"
• "saying I don't know is a powerful thing" '-' humility	• "myths" about the DA program	• "official role"
• "why I take notes"	• "lifespan" for an DA	• "enjoyment"
• being an DA	• "give students time to gather thoughts"	• "rewarding"
• "always learning"	• "giving bad news" is hard	• "seemed like a good fit for me"
• "commitment to education"	• "student reflection on performance":	• "you're not distracted by anything"
• "cool and interesting"	• "teaching during presentation"	• "comraderie"
• "discussing wtih MAs enlightening"	• "when it goes well, not much we can do"	• "variably pleasant and fun vs more challenging"
• "I want them to be good at it" (taking care of pts)	• "describe the process first"	• "draining..emotionally charged"
• "recognizable effort"	• "I do not prepare '-' I show up"	• "grunt work"
• "rewarding"	• "goes well, shorter" vs "doesn't go well,...longer"	• "review all assessments and make recommendations."
• "shared experience" '-' DAs working together	• "role separation"	• "unofficial role"
• "time available unique to DA"	• "I take notes"	• "words matter"
• authentic "real patient"	• "keep my mouth shut" '-' being unobtrusive	• "enlightening and useful"
• DA program needs me	• "tends to end up being subjective"	• "garbage in garbage out"

### Role Enactment- what the DAs do and why

Designated assessors experience predictable, purposeful activities that occur before, during, and after observations and assessments in their work with learners and with one another. The DAs have developed a shared mental model of their role and can easily articulate its key elements. They attribute their understanding of the role to the purposeful professional development required for and embedded in time assigned for the DA position, as well as to their encounters with students and discussions within the Entrustment Committee.

Prior to an observed assessment, the DAs are careful to clear their minds and set potential biases aside: “I have an assessment later today with a student who was on my service in the prior rotation. I obviously know that student to some extent, but I try not to bias myself by other people’s prior opinions of the students. I just show up.” DA1. The designated assessors then “spend a fair amount of time talking to [students] -- before we even start looking at patients -- I spend a fair amount of time describing what we’re going to do and also the rationale for why we’re doing this.” DA3 Additionally, they work to dispel myths that may be passed down by upper-class peers. “There’s a little bit of assessing for presence of those myths and trying to help establish with them that the most important concept is for me to observe them doing…‘authentic work.’” DA1. A designated assessor “…[tries] not to look at a student’s prior evaluations. I do ask them areas that they were told before to work on, or based on their own self-assessment, areas they feel like they’re struggling in.” DA6 Finally, prior to the assessment, the DA tends to logistics such as, “‘Does the attending physician in this clinic, or whatever, know we’re here?’ and, ‘Are they OK with me being in the room with their patient’s permission?’” DA2. They gain this permission, letting the patient know the DA should be treated as though they are a “fly on the wall,” DA5, and telling the student “that unless you’re going to do something that’s going to hurt the patient, I’m not going to say anything.” DA3 Working through these foundational issues helps set the stage for a meaningful assessment.

The DAs physically position themselves out of the patient’s line of sight at the beginning of the clinical encounter and assessment. “…we ask the patient to ignore us as assessors… We want it to be as authentic as possible…” DA6 Since the DAs are not on the care team, and because patients naturally look to the senior care providers in the room, this action ensures that the learner is leading the clinical encounter as part of their authentic work. The intentionally planned and focused mindset of the DAs during the encounter enhances the information collected and feedback provided: “You’re there. You’re a fly on the wall. You’re not distracted by anything. No pages, no texts, no emails. You’re watching the student do that. Your brain is thinking at the same time. Sometimes they come up with questions. You’re like, ‘Whoa, I didn’t think of that.’ Sometimes you’re like, ‘OK, you need to ask this.’ You’re actively engaged in the process.” DA5 Nonetheless, the DAs are challenged at times as the “hardest part about being an [Designated] Assessor…versus a teacher is I have to keep my mouth shut and my facial features totally flat so as not to either, A, influence a student, or, B, potentially upset the patient.” DA3 Another key feature of their experience in the room, which reinforces their non-participation in the patient’s care, is “take[ing] notes throughout the encounter [for each EPA], to ensure that I’ve captured all my thoughts…,” DA6 recording an accurate picture of the student’s skills in that one encounter.

Following the in-room clinical encounter and assessment, the DA may watch the student’s presentation to their attending, resident, or team member. Once the observed encounter and presentation are complete, DAs ask the student, “How did they think they did with the whole encounter. That’s a really marvelous part, getting them to give me their feedback on their own performance. Their self-awareness really comes out in that environment.” DA4 The student’s self-reflection helps the DA frame feedback to be as useful as possible, particularly regarding the student’s previously identified area for improvement. It is critical that “feedback is all at the end…If I give feedback at the end of the history before they did a physical, I might be helping them pick a better physical. That’s more like teaching at the bedside as opposed to observation and feedback.” DA2 “We try to give students very detailed and specific, both in-person and then narrative, commentary.” DA6 Through their experience, the DAs have learned that for students, “the more valuable feedback…is the immediate feedback. [We], sitting face-to-face talking about things, having a discussion about things they did that worked well, things that they may have known didn’t work well, and looking for advice… The written feedback is more useful to us as the EC, but the immediate feedback is the much more useful part to the student.” DA1.

After their engagement with the student, DAs complete necessary documentation to capture the observation and feedback. “When we’re done, then I just give them the instructions on how to get me the…the forms that I have to fill out, and we say goodbye.” DA4 “I spend most of my time writing the assessment…providing constructive feedback, actionable points, and how to fix that.” DA3.

The DAs are also “… part of an EC [Entrustment Committee], which, throughout the year, looks through all the students’ EPAs and evaluations to date. We try to, as a group, identify areas for individual students that they need improvement, but also, hopefully identify students who may be at risk or may need even remediation or just additional coaching or teaching early in the year so that they can fine-tune those skills and be at the same level as their peers. It gives us a very individualized approach to assessing students.” DA6 Further elucidating their EC experience, “Discussing the students as the whole team… is much more enlightening and useful…” DA3, as “Many times a number of people in the room have had an experience with them or knows even more about them. We talk about those things. We go back over the written stuff. We look at our stuff, and we also look at what the residents and faculty have said about them too.” DA4 They experience this EC work as somewhat overwhelming, as “…There’s a lot of stuff to look at, a lot.” DA3 “The process is very different and hard and complex and full of gray.” DA1 “…we review all of that for every EPA that a student’s done throughout the year to try to make those types of [entrustment] decisions. It’s certainly a group that’s doing the work.” The results of these EC meetings are shared with “…the students and the coaches. The students sit down with the coaches with that information we’ve provided as the EC to help direct a course forward in terms of how can we work on those skills throughout the year.” DA6.

### Community Meaning-Making, how a Shared DA Identity and Language are Crafted

Designated assessors value the professional community that they are building together. “We worked a lot, in the beginning, to try to develop core belief systems…about what we expect, what we mean, what we say…so that we are able to…know what each other means. That’s taken, obviously, a lot of time together and working with each other and working through other problems related to the program…” DA4 Further illuminating their experience as a DA community, they feel they “have these moments to chitchat here and there. That’s made me feel a bigger part of the School of Medicine side of things and the student life…I like being part of things that haven’t been done before and building things from nothing…That creative part where we continue to work on the program and decide how we’re going to do it…is very fulfilling to me. There’s the camaraderie of the new team…Others coming from all parts of the big hospital system… That’s been really nice and fun to work together in a way that we hadn’t.” DA4.

Creating a shared mental model to consistently conduct high-quality assessments, the DAs “learn stuff together. We read articles about how to do good assessments and try to get inter-rater reliability (sic) to its maximum, and things like that,” DA2, and “it is like a paint-by-numbers game with all the assessments that come in from these different places. We trust each other’s assessments very strongly. We all recognize the challenges that we all face.” DA4. The DAs strive for consistency, even in the smallest details. “This last meeting, for example, we were told not to write ‘the student,’ but to write ‘you,’ because even though it’s addressed to Jane Doe, Jane may think that somehow that’s detached from her…It’s complex, but it’s more well-understood by all of us now. I still see it as a living…breathing organism that is going to continue to evolve.” DA5 This community also supports DAs’ individual growth as professionals, affording them opportunities to further hone their teaching and assessment skills. “I’ve gained a lot of what I personally wanted to gain in terms of getting better at doing this job. [If we didn’t have the program] I would stop progressing in my own development as someone providing feedback to all the various people I work with.” DA1 The DAs recognize that due to their deliberate standardization in their approach, their data is useful and valuable to decision-making by the EC, and helps clarify data from non-DAs that can sometimes be nonspecific or lack critical details…“The quality of the written content varies dramatically by the individual providing it.” DA1.

The foundation of the DA program is conducting directly observed workplace-based assessments and providing feedback to the students. As long-time clinical educators, however, the DAs experience a natural pull to teach and thus experience some degree of tension about their role. “Constructive feedback is a slippery slope to…[becoming] an educational session. I think the rationale, which I understand and I believe, [is] for us to simply be assessors.” DA3 It appears that the DAs reconcile the tension by adopting “Polarity Thinking” or a “both/and” mindset [35]. “I have evolved into doing a little bit of teaching during the feedback session as well because I want to give them something, especially when I [have] a lot of constructive feedback.” DA2 The DAs universally identify as educators, having “a long-standing commitment and interest in teaching.” DA3 “It’s really about being an educator more than an assessor. Assessment is part of what we do, but we’ve, one, been given the opportunity, and two, the training to have a very unique experience with students…there’s not as much as just dedicated, individualized instruction that you can provide without protected time like this with students. We specifically have this time allocated where we don’t have our clinical responsibilities, so we’re not distracted by that. It’s our opportunity to really help a student fill in the gaps and identify areas to improve.” DA6 This motivates them to be involved in the program. “I have a job to do there, where I need to make objective assessments, but it’s also a supportive role, mentorship role and teaching role, because of the fact that we provide feedback immediately and then later in other written forms. I have the opportunity to not only show the students what I observed and how I feel about that, but also to provide them with opportunities for change that they may not know about already and ways to handle their discomfort about all of it [laughs] as well.”DA4 Interpretation of the DA’s words highlights that, for them, the program reinforces the importance of assessment, feedback, *and* teaching.

### How DAs Experience the Work of Identifying Struggling Learners

Designated assessors are motivated by the opportunity to identify struggling learners so that they don’t fall through the cracks. “We wanted to implement a way for us to capture that small minority of students who we felt weren’t ready … to be doctors yet, who didn’t have the skill or the mindset yet to have our [school] name on their diploma.” DA3 They are part of “rectifying that problem,” noting that “as each quarter goes by, we’re honing a list of people we think need some extra attention in certain areas… Everyone is driven to help the students. We know that it matters. We believe, and heck, these kids are going to take care of me someday, so I want them to be good at it.” DA2 The DAs believe “we’ve made significant headway. I don’t think too many students escape evaluation…We now are much better at detecting who needs help,” DA2 “I would be sorry for the loss [of the program] to student development…We can help students get where they want to go and be better prepared as residents when they get there, because of this program. The kids that we pick up in need of remediation, in the past, many of those would have slid right on through and walked in some places a resident, and then didn’t fulfill the expectations or struggled mightily…” DA4, adding “The students are thirsty for this kind of stuff. They want to be one-on-one with a seasoned clinician at the bedside learning stuff, and we don’t give enough of it to them.” DA2 The DAs “feel like I’m helping them,” DA3, which they find rewarding. “The ones who are struggling the most *need* someone to say, ‘You didn’t do very good [sic] on this. Here’s what I would do differently next time, and here’s how you might approach that.’ I feel like one of the advantages that I have, or all of us [Designated] Assessors have, is it’s a one-time encounter for us. I’m not their ward attending. I’m not spending three weeks with that student. I hit and run. While I definitely want it to be a good experience for them and give as much positive feedback as is appropriate, I don’t have the baggage of, ‘I’m going to have to round with this student in the morning.’ I feel like I have more comfort in giving them the constructive part that I would if they were following me around for three weeks. It’s harder when you’re doing that … You might think, ‘Well, you have a relationship with them, so it should be easier.’ I’m thinking like, ‘My job right now is not to teach you. It’s to evaluate you, so I’m going to do that.’” DA2.

## Discussion

Given the unexamined impact of using a well-trained group of designated assessors for both formative and summative assessment in an EPA-based assessment program, an important first step is to understand the lived experience of such a specialized group. This study sought to illuminate how designated assessors (DAs) *experience* and *make sense of* their role as workplace assessors and as members of an Entrustment Committee. An interpretivist phenomenological approach helped surface structures of meaning that define the DA experience in this setting, deepening knowledge for the implementation of frequent assessors in EPA and other competency-based assessment programs. Designated assessors provide rich descriptions of their processes, shared experiences, the meanings they make of these experiences, and the value they derive from them. Working together, the research team interpreted these data to understand that the DAs find the unique assessor community consequential, and the quality assessment data they provide valuable to learners and those who support them.

Designated assessors experience something akin to a Community of Practice (CoP) in their work together [36], as they share common goals, learn from one another, and support one another. While this resembles a “community of practice,” our data suggest something more specific: the DA group operates as a recognized forum where assessors rehearse, refine, and authorize what “good assessment” feels like from the inside. The affective tone, which reflects camaraderie, responsibility, and pride, suggests that this is also identity work: participants who already identify as educators are becoming assessors together. The community thus functions as a place that holds the tension between educator and assessor identities and lends support to the acceptability of restraint during assessment (e.g., silence during encounters) that might otherwise feel like a relinquishment of teaching. These shared experiences reinforce their collective commitment to providing quality assessment data [37, 38] and to how it is used to inform high-stakes student advancement decisions [19]. Their CoP provides synergistic benefits for the DAs and the EPA program, enhancing their professional growth, improving the assessments they provide, and reinforcing their commitment to their DA work, all of which contribute to program quality.

The designated assessor experiences students’ participation in focused 2-hour DA sessions multiple times across their clinical rotations as highly learner-centered. Participants articulate a persistent tension between the urge to teach and the obligation to assess; a polarity they manage through time-bound teaching (post-observation) and disciplined silence (during observation). Their work to identify struggling learners, however, is seen as a critical responsibility to learners, patients, and the institution’s social contract. The DAs’ special vantage point is experienced as enabling candor: far from daily clinical work, it gives them permission to say what might be hard to say in ongoing learner/faculty relationships. We interpret this as evidence of their commitment to assessment as a mechanism to support students’ growth and development. Balancing their learner-focused perspective does have challenges, though. To manage the uneasiness that comes with high-stakes decisions, participants described relying on the benefits of group decision-making. They found this work on the Entrustment Committee both demanding and reassuring. We interpret this collective approach as a way of spreading the weight of difficult decisions across multiple people and grounding those decisions in reasoning that the group has worked through together [39]. The DA’s shared experience and collaborative creation of decision-making rules can be understood as norms that acknowledge their dual role in providing formative feedback and making summative decisions.

The designated assessor role requires more than clinical expertise; it demands a sustained, intentional presence across multiple dimensions: being in the right places, investing time, engaging directly with learners, and building genuine relationships. Together, these practices signal a commitment to fair and authentic assessment. When designated assessors cultivate trust with learners, they give learners the confidence to be honest about their struggles. When designated assessors cultivate trust with each other, they create a community of support for the difficult work of making judgments about learners’ competence.

Incorporating DAs into our EPA teaching and assessment program has supported a system that meets the criteria of high-quality programmatic assessment [40, 41] and has provided a return on investment for the institution through supporting the growth of individual learners, advancing the skills of the faculty involved, and providing high-quality assessment data that, in aggregate, benefits the institution itself [5]. The DAs articulate the program’s advantages to the institution, believing that it would be a loss if the program were to cease. The aggregate data from the EPA program is crucial for evaluating the curriculum and enhancing existing educational experiences and programs to support students. Early identification of students who are not progressing as expected facilitates appropriate resource allocation for remediation [42]. Data-driven group decision-making results in greater confidence that graduates of the institution are fully competent, residency-ready, and prepared to care for patients [18, 43].

This study has some limitations. This specific program has been implemented at only one institution to date that we are aware of. The DAs applied for these roles and self-identified as educators, which naturally influenced their perspectives. They are clear advocates of the program and, as such, may have offered a positively biased perspective. We are unaware of any substantive experiential differences between participants and nonparticipants. Despite our efforts to remain reflexive throughout, our individual program experiences influenced our interpretation of the data. Despite limitations, this phenomenological study offers an in-depth exploration of this unique assessor role in an EPA program.

## Conclusions

The DAs clearly articulate that their role is viable, important, and makes a difference in the lives of learners and the institution. They know they are identifying struggling students and providing opportunities for them to receive support; this creates pride and satisfaction among DAs. The DAs experience tension related to their assessment role, as these seasoned educators have a strong desire to teach, develop, and correct learners; however, they reconcile this tension through post-assessment teaching without compromising the quality and impact of the assessment. Anticipated role-based tension between formative assessment and summative decision-making regarding student progress is mitigated by the purposeful development of the DAs within their supportive community of practice, and a reinforcing structure of strong assessment processes and results that support defensible advancement decisions for learners. Although the DA experiences shared here are based on a single institution, the EPA assessment program was implemented with fidelity to the core components of EPA [44], making it transferable to other schools. We share these experiences with those developing similar DA programs, ultimately to enhance the quality and impact of clinical assessment.

## Data Availability

The datasets generated and/or analyzed during the current study are not publicly available, as infallible de-identification cannot be assured. However, they are available from the corresponding author upon reasonable request.
